# Development of a Novel Railway Positioning System Using RFID Technology

**DOI:** 10.3390/s22062401

**Published:** 2022-03-21

**Authors:** Osama Olaby, Moussa Hamadache, David Soper, Phil Winship, Roger Dixon

**Affiliations:** 1 Birmingham Centre for Railway Research and Education (BCRRE), School of Engineering, University of Birmingham, Birmingham B15 2TT, UK; m.hamadache@bham.ac.uk (M.H.); d.soper@bham.ac.uk (D.S.); r.dixon@bham.ac.uk (R.D.); 2 Network Rail, Asset Enhancement Team, Milton Keynes MK9 1EN, UK; phil.winship@networkrail.co.uk

**Keywords:** railway, track switch and crossing, RFID technology, positioning system

## Abstract

Currently, a number of positioning systems are in use to locate trains on the railway network; but these generally have limited precision. Thus, this paper focuses on testing and validating the suitability of radio frequency identification (RFID) technology, for aligning vehicles to switch and crossing (S&C) positions on the railway network. This offers the possibility of accurately knowing the position of vehicles equipped with monitoring equipment, such as the network rail track recording vehicle (TRV), and aligning the data with reference to the locations of the S&C (and ideally to key elements within a particular S&C). The concept is to install two tags, one on the switch-toe sleeper and the second on the crossing-nose sleeper, with an RFID reader that will be installed underneath the vehicle. Thus, the key features of the S&C, the switch toe and crossing nose, will be considered as a definitive reference point for the inspection vehicle’s position. As a monitoring vehicle passes over a piece of S&C, the proposed positioning system will provide information about this S&C’s ID, which is stored inside the RFID tags and will indicate the S&C’s GPS coordinates. As part of the research in this paper, more than 400 tests have been performed to investigate two different RFID technologies, passive and semi-passive, tested in a variety of conditions: including different passage speeds, different distances between the RFID reader and the tags, and varied strength signal transmitted between the reader and the tags. Based on lab testing and analysis of the recorded data, it is concluded that passive RFID technology is the most suitable of the two technologies. The conclusions find that the proposed RFID-based solution can offer a more precise positioning solution to be a reference point for the train location within the network.

## 1. Introduction

### 1.1. Literature Review and Reserch Gaps

Automatic train operation (ATO) requires that the position of the vehicle in the railway network and its speed be accurately determined [1,2]. Furthermore, to ensure train safety, positioning a vehicle is an important task to be solved, which has resulted in the development and use of several railway detection/positioning technologies. Many of the already implemented technologies can only detect the vehicle within a defined track range. For example, track circuits are used for signaling purposes to ensure the track section is clear to allow the next train to pass [3]. This traditional method suffers from the change in resistance between two devices. This can be due to wet track, resistance changes between the rails, broken rails, and wheel-rail contact problems. Additionally, the track circuits method cannot identify the train running between them, but only ensures that a track section does not have a train. Another primary detection method, but also not an identification technique, are “axle counters” that consist of wheel sensors mounted at each end of the track section and an evaluation system that counts the number of axles that pass in and out of this section [4,5]. In a different detection method, using lineside acoustic equipment [6], a train’s vibration pattern can be sensed which can detect the presence of a vehicle and its speed on the track. In an advanced solution, but one that has not yet been deployed, trains are provided with a camera facing the running direction. The recorded images can be compared against the reference images in a database to locate the position of the vehicle in the network [7]. A similar, but alternate approach to looking into camera images, could be the development of barcode-type images to be scanned and recognised by algorithms to minimise the amount of data to process [8]. Furthermore, an integral part of the European Train Control System (ETCS) is the Eurobalises [9,10], that act as a transponder when are placed between the rails on the sleeper. They transmit information similar to a telegram to a passing train which helps to identify the exact location, as well as read any signaling information. Although these Eurobalises are useful for locating trains (the position uncertainty is given with typically ±5 m [11]), the technology will be relatively expensive when installed throughout an entire network [1]. In a commercial system, which is used and installed in the Czech Republic on heavy-rail tracks, specific balises were mounted on sleepers, and wheel-counters are used for relative positioning on the locomotive, with the precision given to within ±2 m [12]. Though using commonly available GNSS signals is a promising approach to figure out a vehicle’s location and then transmit it to the driver or control unit, higher accuracy is needed to give an accurate train’s position [13]. Additionally, GNSS systems do not work in covered areas and tunnels. Thus, wherever signals are lost, additional equipment, such as gyros and accelerometers, will be needed to improve position resolution using inertial navigation [2], which will make the positioning system more complex. Thus, the limitations in all currently used location systems, including GNSS-based positioning approaches [14], have led to this current research.

Research projects including In2Track [15,16], In2Smart [17] and TfL-London Underground AIT project [18] suggested that Radio Frequency Identification (RFID) technology can be a suitable candidate for developing an accurate train positioning system. This is because RFID has numerous advantages over other systems when carefully matched to the requirements of the application [19]. The RFID equipment can operate in all weather, is robust, has a range of several meters and does not require a line of sight. A deep literature review reveals that the different types of this technology (passive and semi-passive) has never been investigated for accurately positioning railway inspection vehicle. In the railway, RFID systems have been mainly used to facilitate infrastructure asset catalogues and automated tracking/detection of trains [20,21]. Through the Horizon2020-In2Track project [15], a comprehensive list defining the suitable wireless communication to develop a positioning system and its architectures were described and studied. Another piece of work has been presented in the In2Smart project [17] where a demonstrator of identifying vehicles with passive RFID tags has been developed. As a part of this demonstrator, RFID readers were installed in four locations near the track which correctly identified 94.4% of 798 trains during 180 days. The In2Smart work concluded that the demonstrator paves the way only for a standalone automatic vehicle identification system but not for train positioning purposes. However, in the present work, In2Smart’s concept has been extended where the infrastructure components have been identified by an RFID reader, which is located on a maintenance vehicle. Moreover, in the presented research, roles are flipped, as will be presented in Section 2, since the transceiver (RFID reader) will be mounted in the vehicle whereas the transponder (RFID tag) will be located in the asset of interest, i.e., the switch/crossing unites.

Currently, the only deployed RFID system in a real environment for localisation of vehicles on the track is on the London Underground (LU) network, where semi-passive RFID technology [22] has been installed for Automatic Track Monitoring (ATM) purposes [18]. The installed tags [23], which have a battery life of around 8 years and are mounted to the sleeper, have been used to accurately define the vehicle’s location on the LU network and therefore accurately report the location of faults as they are discovered. Though these semi-passive tags are accurate to ±0.5 m at vehicle speeds up to 40 mph, LU engineers have realised that there is a clashing of the RFID reader frequency with other equipment such as the Communication Based Train Control (CBTC) system [24], radio change beacons and even Wi-Fi.

Presently, recording vehicles are unable to align the data they collect with any information about the switches and crossing being traversed: such as their ID, location on the network, or location within the S&C of key elements (such as crossing nose, switch toes, etc.) [25]. Although (in the UK) the Track Recording Vehicle (TRV) uses linear referencing, presented by mileposts [26], to calibrate its position on the track and has recently used the GPS, the current positioning system is not accurate enough to reference the maintenance data to the key features of the S&C, such as the switch toe and crossing nose. Determining the accurate position of the TRV in relation to an S&C is still a challenge.

### 1.2. Contributions, Novality and Reserch Goals

As a potential solution to the problem of the frequency clashing of the semi-passive RFID technology with other equipment when this technology has been used in LU [22], this research paper aims to investigate and test another type of RFID technology, and then compare its performance with the semi-passive RFID. This is the first main contribution of this work which aims to develop the use of RFID devices, with application to positioning of any vehicle equipped with monitoring and measuring equipment such as the TRV. Thus, the novelty of this research lies in using, for the first time, a passive RFID technology (which is primarily developed to identify objects) for accurate positioning purposes that will be able to improve the reliability of the TRV in inspection tasks.

Further, implementing an identified node (i.e., RFID tag), that can be used as a definitive reference point for the location of the TRV in relation to the S&C on the track, will ideally be harmonised across all Network Rail databases. This output could also help to pinpoint the exact location of the track geometry defect [27], measured by the TRV, in relation to a key part of the S&C to show the accurate position of a faulty S&C in the output maintenance report of the TRV. This improvement in the track-geometry-defect reporting will help to direct the maintenance staff to the exact S&C that requires maintenance. This improvement of the TRV inspection-reporting system can be considered as the second main contribution of this paper.

Therefore, the proposed train positioning solution in this paper has two main aims. The first is to improve the position accuracy system within the railway applications, which is currently limited to ±2 m. This will be through the exploitation of using an RFID tag, installed on the track, to be a definitive reference point for the inspection vehicle’s position. The second aim is the ability to improve the TRV inspection-reporting system, especially for the track-geometry-defect output maintenance reporting. The two RFID technologies proposed to be the main part of the positioning system, passive and semi-passive RFID [19], have been investigated in railway laboratory testing. Then, an evaluation of the performance of each RFID technology has been carried out based on analysis of the experimental data recorded during the testing works.

A deep analysis of the results shows that the proposed novel positioning solution based on the RFID technology has achieved a position accuracy of less than ±1 m, which is double the best accuracy that can be achieved currently. This has a very significant impact on better planning the railway network and ultimately helps increase its capacity. Further, the test results show that this high accuracy can be achieved at low, medium and even at reasonably high-speed cases. This has been confirmed by testing the passive RFID technology at a speed of more than 100 mph and the semi-passive technology at a speed of around 70 mph. One important observation is that depending on the speed of the passing locomotive (tag(s) in the current demonstrator), an adequate signal strength, transmitted between the RFID reader and the tag, should be adjusted to meet this targeted high positioning accuracy of less than ±1 m. This important finding can be considered the third contribution of this paper. A recommended interval of the adequate signal strength for each speed range (low, medium and high) has been proposed in this paper, where more specific values for more defined speed values will be the next aim of this work.

In the present paper, the developed demonstrator of the positioning system and details the building of an experiment rig to facilitate the testing of the RFID technologies performance are presented in Section 2. This section also describes the testing and comparing of two types of RFID technologies for further evaluation in terms of system performance and repeatability. The experimental results, in a lab environment, of tests of the main sub-system, are presented and discussed in Section 3. Finally, conclusions are drawn and some ideas for future work are given in Section 4.

## 2. Materials and Methods

### 2.1. Positioning System Overview

**Figure 1:**
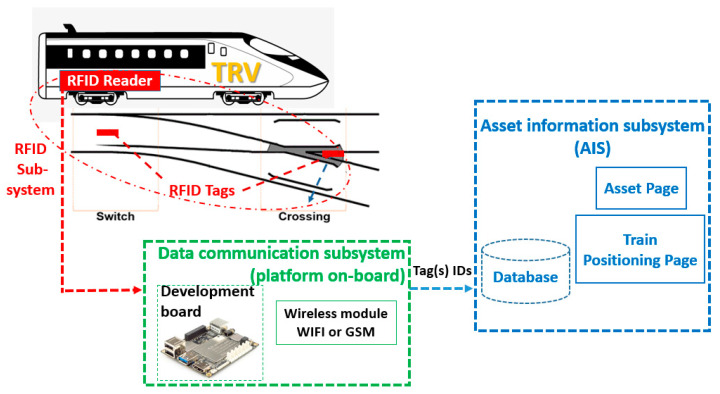
Layout of the complete positioning system, including the RFID subsystem, the data communication subsystem (DCS) and the asset information subsystem (AIS).

In this research work, RFID technologies will be used as the main part for developing a railway positioning system and thus correlating the S&C’s position to vehicles, specifically to TRV, for alignment of position and data information, and position of the asset for maintenance. The whole proposed system consists of three sub-systems, the RFID subsystem, the data communication subsystem (DCS) and the asset information subsystem (AIS) as is shown in Figure 1.

The RFID subsystem includes mainly two parts, the RFID transceiver (reader) and the transponder (tag(s)). The idea behind this work is to install two tags, one on the switch-toe sleeper and the second on the crossing-nose sleeper, with an RFID reader to be installed underneath the vehicle. Each tag ID will be replaced by a real switch or crossing number. The tags do not hold any other data as this can affect the accuracy of the positioning when being transmitted to a vehicle through the reader, due to the time to transmit longer data strings. The key features of the S&C, the switch toe and crossing nose positions/GPS coordinates, will be considered as a definitive reference point for the inspection vehicle’s (i.e., TRV) location on the track. When the TRV passes over a S&C, the positioning system will provide information about this S&C’s ID, which is stored inside the corresponding tags, within the railway network. This will be achieved by the RFID reader communicating with the tag(s) and reading the S&C ID that refers to its position within the rail network.

The data communication subsystem (DCS) is responsible for transferring the data recorded in the RFID tag to the Asset Information System (AIS) where all the history maintenance information will be stored. The DCS platform will include a developed electronics board (e.g., LattePanda [28]) that interfaces with the RFID reader. It will use either GSM or WIFI technology so that the information can be uploaded to the AIS database. Thus, through the GSM (or WIFI) network, the on-board positioning system will be able to report the tag ID recorded by the TRV. These tag IDs stored in the AIS database will indicate the S&C’s GPS coordinates.

This paper focuses on describing the development and testing of a demonstrator of the first subsystem, the RFID subsystem. The second and the third subsystems, i.e., the DCS and the AIS subsystems, are not included in this paper and are to be considered for future works.

In the current work, two RFID technologies were proposed and investigated. The first is based on passive technology, whereas the second is based on semi-passive technology. Both technologies’ features and characteristics are selected to meet the requirements of the positioning system that have been identified in a technical stakeholder workshop, where six experts from different disciplines in Network Rail (NR) have participated. All participants were in the position of Principal Engineer and from six different departments within NR, including Intelligent Infrastructure, Safety Technical Engineering Directorate, Mobile Monitoring, Data Analysis, Technical Authority, and Asset Enhancement.

The selected passive RFID technology includes the RFID Speedway Revolution reader: R420 from Impinj [29] and a RFID panel antenna “Laird-S8656XRRN” [30]. R420 supports a separate read and writes process operating at a power range that starts with 5 dBm to +31 dBm (command-adjustable). The used frequency range starts from 860 to 960 MHz to accommodate worldwide regulations [31]. The “MultiReader” software can be used to read/write from/to RAIN UHF RFID tag “Confidex Ironside”. Additionally, specific applications to command the R420 embedded module can be written using high-level API which supports C programming environments.

For the semi-passive RFID technology, the “TRANSIT Ultimate” reader [23] is selected, tested and compared with the above passive RFID-Speedway. The TRANSIT has been used in recent years by the Asset Inspection Train (AlT) to define vehicles’ location on the London Underground (LU) network [18]. Hence, it was proposed to test the TRANSIT reader accessories including the semi-passive tags (Heavy Duty TagISO). The semi-passive TRANSIT system has mainly two key features, the first one is its ability for programming with a high-security level which includes encryption of the data transmitted from its tag (Heavy Duty TagISO) to the reader. The data encryption will prevent errors/misreads/false triggering by other types of tags. Therefore, the tags will only respond to the readers they are programmed to respond to. The second feature of the TRANSIT technology is that it is a high-frequency technology (2.45 GHz). This makes it a robust technology. This robustness is partly due to having a stronger signal transmitted between the reader and the tag. Thus, again, the semi-passive tags will only respond to the reader they are programmed to respond to. The full technical description of the semi-passive TRANSIT technology can be found in [23,24]. The more suitable RFID technology, for the application in this work, among these two will be highlighted in Section 3.

### 2.2. The Developed Demonstrator

**Figure 2:**
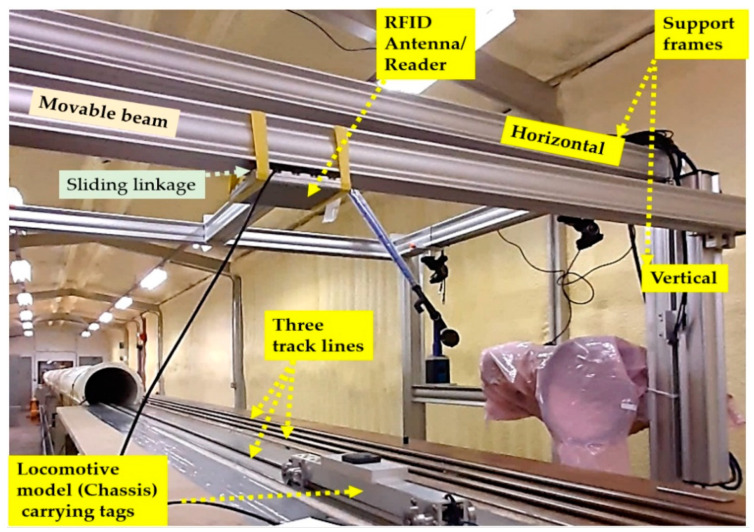
A descriptive photo of the experimental demonstrator.

**Figure A1:**
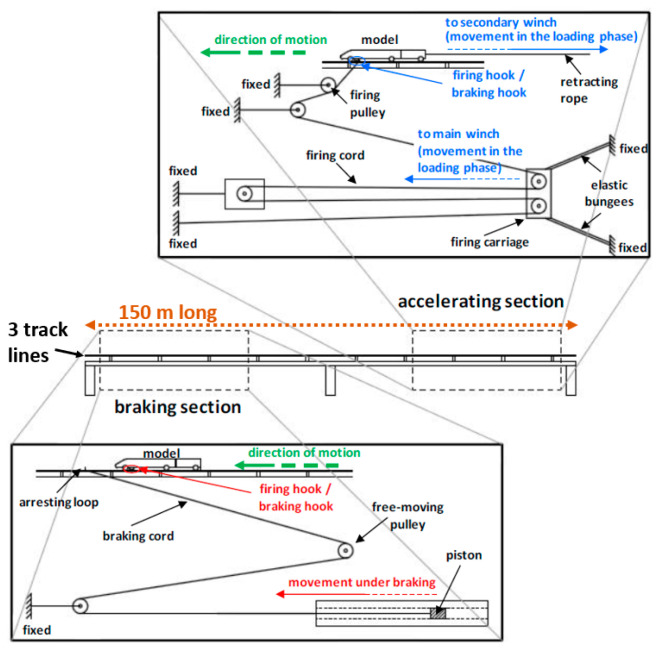
Plan view of the implementation environment showing the locations of the catapult, and the breaking/acceleration sections.

The formal testing phases of a physical demonstrator developed in this work have been carried out in the University of Birmingham TRAIN (Transient Railway Aerodynamics Investigation) rig [32]. This railway testing lab consists of three 150 m long track lines (see Figure 2) along which model vehicles can be propelled [33], in both directions, at speeds of up to 178 mph. Figure A1, in the appendix, presents a plan view of the implementation environment showing the locations of the firing equipment, and the breaking/acceleration sections.

**Figure 3:**
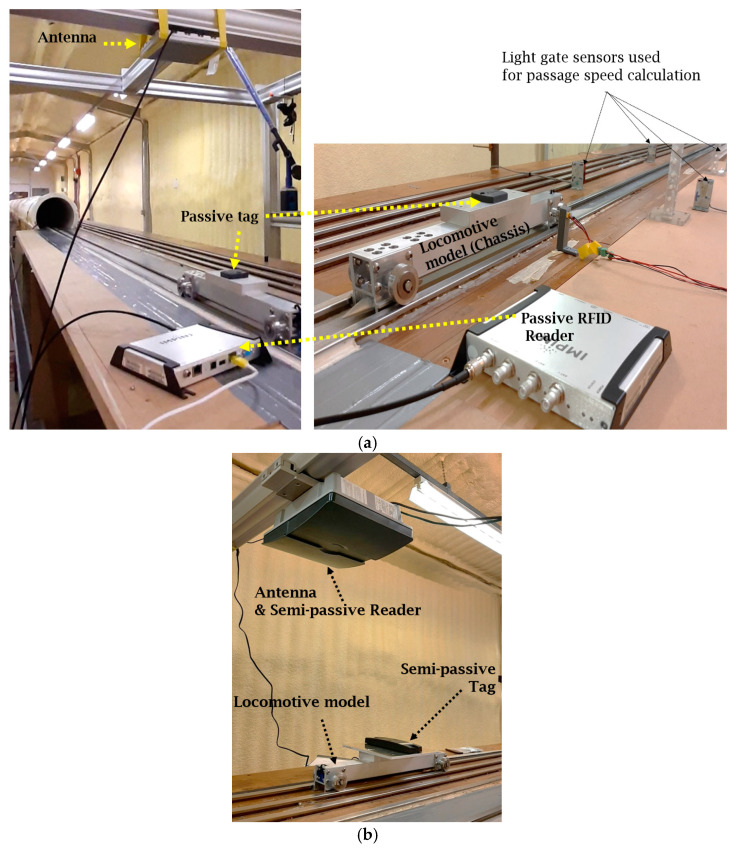
A close-up view of the RFID antenna/reader and the tag(s). (**a**) Passive technology arrangements; (**b**) Semi-passive technology arrangement.

For the testing scenarios and vehicle size limitations, the RFID tags were mounted on the small-scale vehicle model whereas the RFID reader was installed on a support frame. To achieve the RFID sub-system testing, many conditions and scenarios were considered including different distances between the RFID reader and tags, provided by a vertical support frame with a movable beam, shown in Figure 2, that can be adjusted to provide a mechanism that allows adjusting the vertical height of the antenna/reader to the tracks. This was essential to meet the system requirements that will be presented and discussed in Section 3. Several customised mechanical linkages were built that allow the attachment of either the antenna (for the passive RFID-Speedway technology as shown in Figure 3a) or the semi-passive RFID-TRANSIT reader (Figure 3b), and also allows the adjustment of its horizontal position to be aligned straight on top of the tag(s) when switching between track lines as shown in Figure 2.

**Figure 4:**
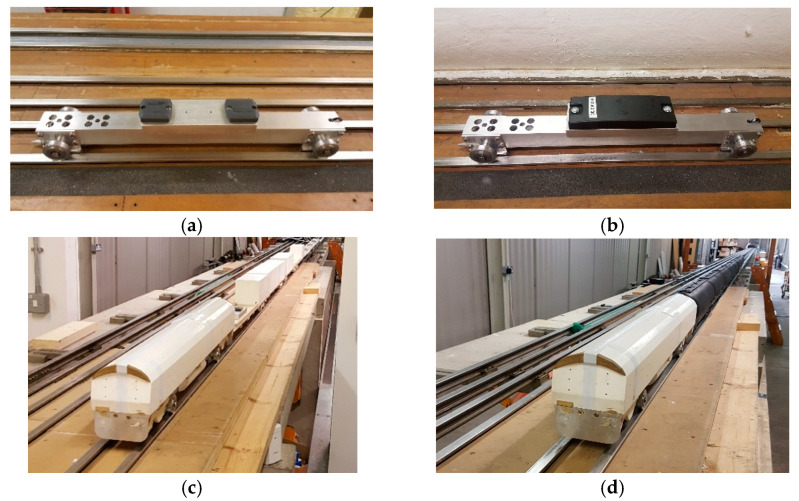
The different carrying model vehicles and their attachments used in the testing lab. (**a**) Passive RFID tags attached to a small chassis; (**b**) Semi-passive RFID tags attached to a small chassis; (**c**) Two passive/semi-passive RFID tags attached to a long model vehicle (tags are uncovered); (**d**) Two passive/semi-passive RFID tags attached to a new long model vehicle (tags are covered with a plastic wagon model kit).

As can be seen in Figure 4, two types of model vehicles were used, a small chassis (Figure 4a,b) and a long model vehicle (Figure 4c,d). The small chassis was used on the low-speed scenario since it is light weight (i.e., less friction), allowing for manual operation on one side, and to achieve a very high-speed when using the firing mechanism, which means that it was also used when testing under the max possible speed (>100 mph). Two passive tags were mounted on top of the chassis with a 150 mm distance in-between (Figure 4a) during each test, whereas only one tag in the case of the semi-passive technology (Figure 4b). The long model vehicle (Figure 4c,d) was used at medium and high speed. Its long length (over 6500 mm) provided an exemplary testing scenario to emulate the installation of two tags with reasonable distance in-between (one at the switch-toe and the other at the crossing-nose). Further, it allowed mimicking the possibility of the tag(s) being covered with a plastic material (Figure 4d) or uncovered (Figure 4c), which could be the case in the real environment, i.e., presence of physical barrier(s) or debris. Moreover, based on the inputs from the stakeholders workshop [30], which has focused on defining the system requirements, the two RFID technologies selected can run in heavy-duty conditions, which includes an ambient working temperature from −27 °C to 60 °C. Furthermore, regarding the vibrations and the humidity conditions, the two selected technologies can meet the requirements set of the conformance standard IP67, where the allowance humidity variation is from 5% to 95% and the acceleration limit for track-mounted equipment is 240 g, as stated in the technical specification of the two technologies [23,31]. This alignment between the technologies’ features and different weather conditions of the railway environments is essential to translating the outcome of this research to the real world.

### 2.3. Testing Plan and Accuracy Calculation Method

The tests have successfully demonstrated the feasibility of the proposed RFID subsystems, the passive and semi-passive technologies, to provide a better possible accurate positioning solution of the locomotive within the network. The locomotive’s position will be in relation to the S&C location, and especially to its key features, the toe and nose. The detection/reading capability was recorded at each performed test and the accuracy values were then generated for both technologies.

The challenges that have been faced while using this RFID positioning system were: RFID reader distance range, strength signal transmitted between the two parts of the RFID technology and the effect of vehicle speed on the reading process. Experiments, especially, on these three parameters have been described, discussed, and demonstrated. Thus, initial different testing scenarios were planned depending on the running speed, the vertical heights, the signal strength, and the presence or not of debris, as follows:

- Speed: (at 400 mm vertical height and 100% signal strength without any debris)
  - low-speed: 5, 10, and 20 mph;
  - medium-speed: 25, 35, and 50 mph;
  - high-speed: 70 mph and
  - max-speed: higher than 100 mph and lower than 120 mph
- Vertical height: 300 mm and 500 mm. Each at high speed (70 mph)
- Signal strength: 30% and 60%; each at high speed (70 mph)
- Presence of debris (leaf of tree): run two times, each under the max speed (70 mph)

These testing scenarios were repeated for each technology and each test was repeated five times to check the consistency. A testing results table and post-analysing are carefully prepared and filled on the testing site. The table contains mainly data about the testing room temperature and humidity, the test number, the considered vertical distance, the planned speed case and the actual speed recording, the detection/reading note, the considered signal strength, the tag numbers, and the firing method (manual or by machine)., It also contains additional columns for data post-processing.

**Figure 5:**
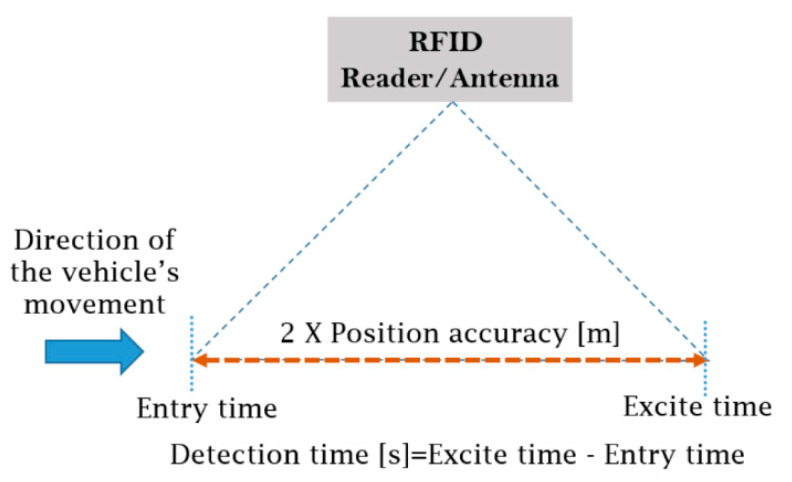
Calculation method of the position accuracy.

**Figure A2:**
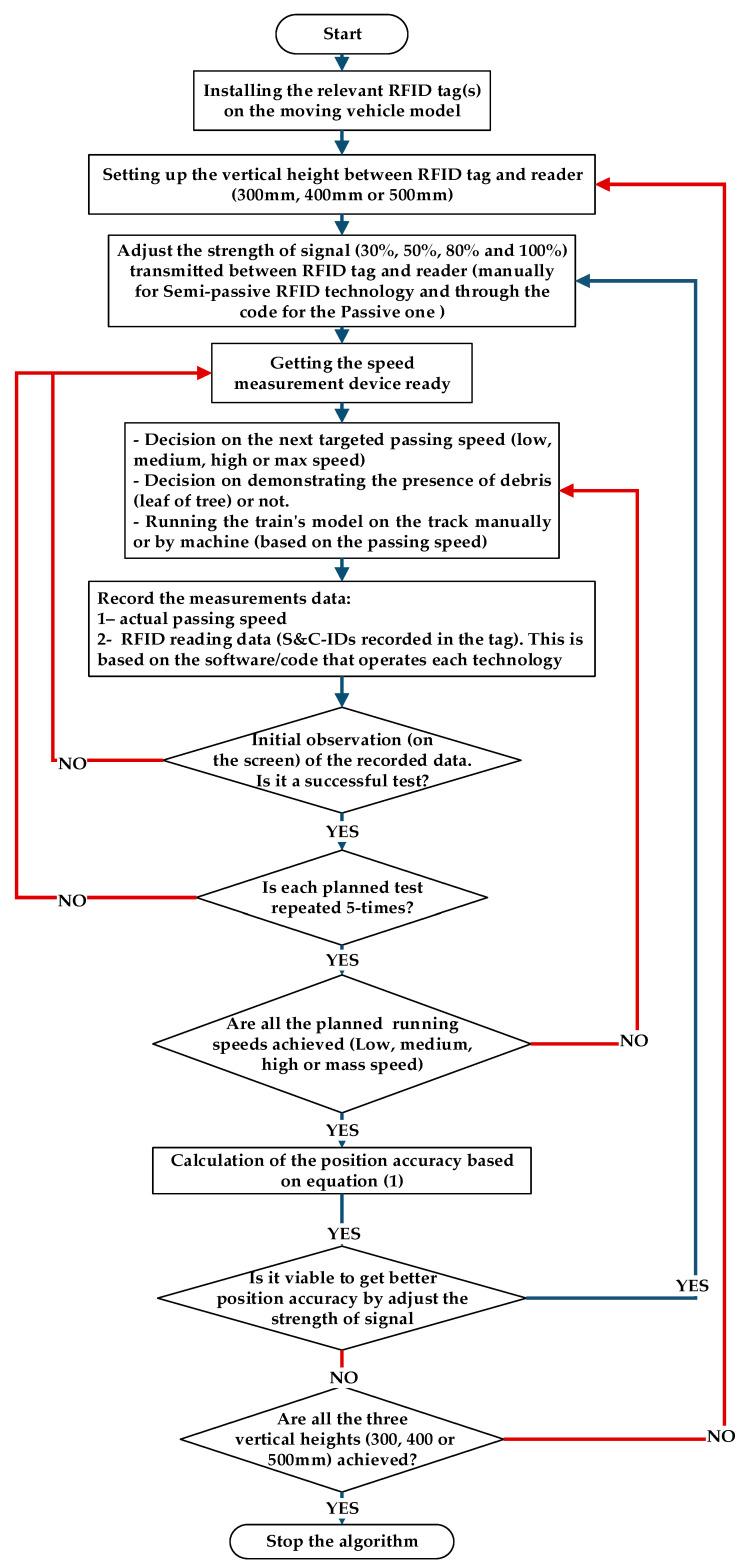
Flowchart describing the measurement’s algorithm and position accuracy calculation.

Several extra tests and scenarios were performed, including low-speed with 300 mm and 500 mm vertical height. This was in order to check if the change in the vertical height (±100 mm) will affect the detection/reading capability and/or the positioning accuracy. Further, different signal strength cases were added (50% and 80%) after post-analysing the preliminary results which are needed to check the impact on the positioning accuracy. In addition to including the max-speed scenario (>100 mph). This made a total of 400 tests that were conducted at the testing site. The flowchart of the testing methodology, for both RFID technologies installed and tested, is presented in Appendix A (Figure A2). For each test, a post-analysis of the results was performed, and the positioning accuracy was generated using the Equation (1) and based on the method shown in Figure 5.

(1) $$\mathrm{Position}\;\mathrm{Accuracy}\left[m\right]=\frac{\mathrm{Vehicle}\;\mathrm{speed}\left[m/s\right]\times \mathrm{Detection}\;\mathrm{time}\left[s\right]\;}{2}$$

The “Vehicle speed” was measured by two sets of light gate sensors installed on the two sides of the testing tracks as shown in Figure 3a. The “Detection time” period, in which the vehicle is visible/detectable by the RFID antenna, was calculated based on the data recorded from the software/code that operates each technology. This period equals to the difference between the exit and entry time of each tag within the RFID antenna range (see Figure 5).

### 2.4. Testing of RFID Subsystem Based on Passive Technology

**Figure 6:**
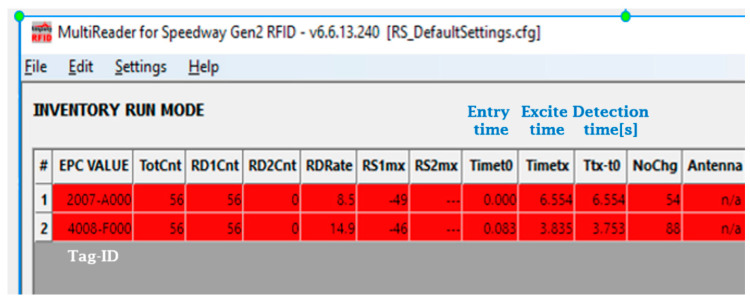
The Speedway software, the MultiReader, interface screenshot.

The passive tags were programmed with real S&C-IDs. The programming step was achieved before mounting those tags on the vehicle model and before setting up the antenna/readers on the support frame in the testing laboratory (see Figure 2, Figure 3 and Figure 4). Two passive tags have been used, the first tag is allocated an ID of “2007A000”, and the second with “4008F000”. The passive tag detection/reading was recorded initially using the MultiReader software, an interface screenshot of which is shown in Figure 6. The MultiReader software also has the option to change the signal strength as needed to perform an accurate position reading.

**Figure 7:**
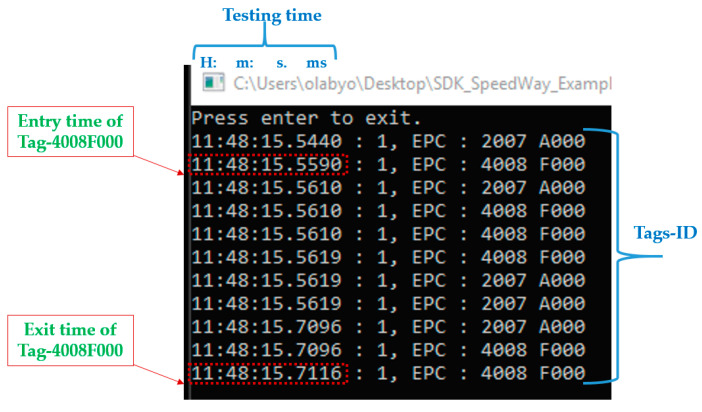
The developed C# program interface screenshot.

After recording and collecting an initial set of data, the preliminary results were post-analysed and found to be inconsistent. It was observed that the entry time to the antenna reading range and the exit time of two tags of the same type was different by a large margin. As can be seen in the two red lines in Figure 6, the entry time (Timet0 of front tag “2007A000” was 0.000 s followed by the entry of the 2nd tag “4008F000” at 0.083 s. This is correct considering the distance between the two installed tags and the passing speed. The exit time of the Tag-2007A000 was at Timetx = 6.554 s, which is within the expected range, but the exit time of the Tag-4008F000 was at Timetx = 3.835 s, which is too short and a cause of inconsistency in the recorded results, especially in relation to the position accuracy. Subsequently, a customized software was then developed and used to operate this technology, written in the C# programming platform. A screenshot of its interface is shown in Figure 7.

As it can be seen in Figure 7, the detection/reading of the two tags, Tag-2007A000 and Tag-4008F000, was consistent. The entry time of both tags and their exit time were in line with the testing conditions including the vehicle passing speed and the C# execution period. Thus, the developed customized C# program significantly did improve the consistency of the data collected, the feasibility analysis capability and helped a better evaluation of the passive technology.

### 2.5. Testing of RFID Subsystem Based on Semi-Passive Technology

In terms of hardware setting-up, the semi-passive Nedap TRANSIT technology was different from the passive Speedway one in two aspects. First, the reader and antenna were both integrated (Figure 3b) compared to the Speedway, where it was separated as shown in Figure 3a. The second difference is that the passive tags were all programed in the same way, but the semi-passive tags were of three programmed types as follows:

1. Tag-N200701: programed with 6 decimal numbers (short message) as a tag-ID, but with a normal speed to sending the ID.
2. Tag-DD000001: programed with 6 decimal numbers as a tag-ID (short message) and also has the possibility of sending the ID two times faster than the normal speed.
3. Tag-00002007AN: programed with 10 decimal numbers (long message) as a tag-ID, but with a normal speed to sending the ID.

**Figure 8:**
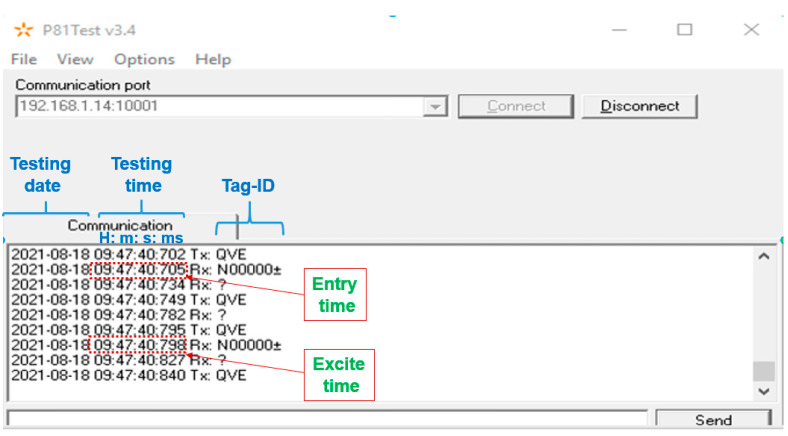
The semi-passive TRANSIT software “P81Test” interface screenshot.

This difference in the programing type was to try to find the best performance tag that will meet the position accuracy in the railway positioning systems requirements as it is defined in a recent study [34] and also in a technical meeting. According to the specification of each tag type, it is anticipated that these tags will perform in the following order (best to worst): Tag-DD000001, Tag-N200701 and then Tag-00002007AN. After testing each type of the semi-passive tags, the results showed that the Tag-DD000001 type was the most promising one, which aligns with the expectation according to the characteristic of each. Thus, all consequent tests were performed using this type of semi-passive tag, i.e., the Tag-DD000001 one. The semi-passive TRANSIT software “P81Test” was found to work well and did provide consistent results. A screenshot of its interface is shown in Figure 8. The software provides the detected tag(s), which appears as its pre-programmed information (i.e., N00000±), the tag entry time to the reader range and its exit time, etc. This information is used to generate the detection/reading capability and the feasibility analysis of the position accuracy based on Equation (1) and Figure 5.

Similar to the passive technology testing plan, the semi-passive testing was performed following the step-by-step testing plan presented above in Section 2.3, which was carefully prepared to maximize the feasibility analysis capability. The data analysis summary and concluding remarks of both RFID technologies will be highlighted in the following section.

## 3. Results and Discussion

Completing all the planned tests, the recording, the filling of the testing results table, and performing post-analysing, a plausible picture of how the two proposed RFID technologies performed was able to be accomplished. The full set of the results and findings with the recommendations are summarized in Table 1. Followed by the observations (based on these results) that are summarised in Table 2.

Based on in excess of 400 tests, it can be concluded that both technologies, passive and semi-passive, could detect/read the tag(s) at low, medium, and high-speed scenarios (i.e., speed from 5 to 70 mph). In addition, both technologies have provided a positioning accuracy of less than ±1 m with the recommended signal strength (see Table 1). It should be noted, as highlighted above in Section 1, that this achieved position accuracy of less than ±1 m is double the best accuracy that can be reached using currently applied technologies. Further, this achieved when positioning accuracy aligns with the requirement system that has been presented in [34] and also has been defined, discussed, and agreed upon during a technical meeting with six expert engineers from different disciplines in the railway engineering sectors [29].

The main difference is that the passive RFID-Speedway was able to detect the passing vehicle carrying two passive tag(s) at a max-speed scenario, from 100 mph up to 140 mph (see Table 1). Thus, the passive technology could provide position accuracy of less than ±1 m even at this (very) high-speed scenario with suitable signal strength. However, the semi-passive RFID- TRANSIT technology could only provide an accuracy of less than ±1 m up to a speed of 70 mph. This could not be a problem within the scope of this phase of the current work since the target TRV speed requirement is set between 5 and 70 mph. However, this would be an issue if this RFID system will be mounted on the Network Rail New Measurement Train (NMT) [35] that can reach a running speed of up to 110 mph. Both technologies did perform similarly when debris was added (either as a leaf on top of the tag or provided by the covered new long model vehicle shown in Figure 4d), where this presence did not affect the detection/reading capability nor the positioning accuracy. This was also similar concerning the change in the ambient temperature and humidity (within the same season). It should be noted that the change in the vertical distance, between the RFID reader and tag, did not affect the detection/reading capability of both technologies. Additionally, the change in the vertical high did not affect the positioning accuracy when testing the passive RFID-Speedway technology. However, for the semi-passive RFID- TRANSIT one, it was observed that when the vertical height changes, the positioning accuracy changes, with a larger height and less positioning accuracy and verse versa.

Table 3 presents an evaluation summary of the two RFID technologies proposed and tested against the most important system requirements, defined at the early stage of this research, and other important specifications defined after having installed and tested the technologies. All nine aspects presented show that the passive RFID-Speedway technology is the most suitable technology for developing an accurate positioning system on a railway track and was shown to outperform the semi-passive RFID-TRANSIT system. For example, the former technology allows the tag(s) to be detected/read using a handheld device, but the latter does not allow it. The passive technology does not clash with other nearby frequencies, but the semi-passive signal can have interferences, especially if the nearby equipment has the same frequency band. Another important difference is that the passive technology has the option of an embedded system supports, SDK, that allows a customised data communication platform development, which allows the integration with the other two subsystems (the DCS and the AIS subsystems) to build the complete positioning system shown in Figure 1. However, this may be impossible with the semi-passive technology as no certain information was provided from the supplier. Additionally, in the passive RFID technology, the fact there is no need to power the tag can make this type of RFID system a good solution to be used in railway track applications as the battery-free tag is expected to be mounted on sleepers. However, from a working hypotheses point of view, using a battery-free tag in the passive RFID technology can be an advantage and disadvantage at the same time for an application. It is an advantage of using a battery-free tag (i.e., a passive technology) because it is expected to operate for an unlimited lifetime. However, the disadvantage is the delay in the wake-up of this transponder every time the reader will pass over it for collecting the data. Thus, in the passive arrangement when the reader comes towards the tag, it has to build up enough stored energy to be able to wake up and send back a message to the reader. So, the passive tag is expected to take a random time (probably within a range of several seconds) to charge up. The random amount of time depends on when the last train passed, how much energy was used and how much is still in the tag’s capacitor. Therefore, in the train positioning system, when the asset management wants to know the vehicle position over the S&C, it may be difficult to get a precise location (within the uncertainty band due to charge time). However, the experimental results recorded indicated that using passive technology-Speedway will not be affected by waking up delay and consequently the free-battery feature will not prevent the passive RFID technology to be suitable for accurately position system in the railway.

In addition to the above points that are in favor of the passive RFID-Speedway technology, several other positive points also were evaluated. This includes the possibility of changing the sensitivity (or the power signal) with software commands or codes on the Speedway technology and not on the semi-passive RFID-TRANSIT one. Moreover, the possibility of the Speedway transceiver (reader) to write to its transponder (tag) and these tags are re-writable, which is not the case with the TRANSIT transceiver/transponder(s). In addition, the Speedway reader mounting place can be allocated a safer location, as only the antenna needs to be mounted underneath the vehicle since the antenna and the reader are separated, contrary to the TRANSIT where both (reader and antenna) are integrated into the same hardware safety case. Finally, the cost of the passive hardware (tags, reader, and antenna) is much cheaper compared to the cost of the semi-passive hardware.

Thus, in the point of view of tag(s) detection/reading and the positioning accuracy within the defined “System Specification”, both Speedway and TRANSIT subsystems could provide and achieve a promising result. However, in terms of “Top-level requirements” including, functionality, data communication, usability and practicability, and costs, the passive RFID-Speedway technology is more suitable than the semi-passive RFID-TRANSIT technology.

## 4. Conclusions and Future Work

The research presented in this paper provides an insight into the use of a modern wireless identification system—RFID technology—for accurately positioning any railway vehicle that is collecting data on the track. A lab demonstrator of the proposed system has been built and tested to prove the principle of using the RFID technology at different speeds for accurate positioning. This lab demonstrator gives an overview of the accuracy of railway positioning systems based on two different RFID technologies, passive and semi-passive ones. Thus, laboratory testing of the main subsystems of the demonstrator has been described. Then, comments on data analysis of the recorded data have been presented. Based on this analysis, an evaluation of the performance of the physical demonstrator has been carried out. It has been concluded that the passive RFID technology is the most suitable solution for the positioning system developed.

Conducting the numerous tests within this phase of research work, the selected RFID subsystem, the passive RFID-Speedway, will feed directly to the next phase of this research work within the Shift2Rail-In2Track3 project [36]. This will be targeted with an attempt to achieve a possible higher positioning accuracy. It is expected to be double the position accuracy recommended in this paper (±1 m) by defining the right operating conditions. This could be by developing an adaptive RFID system in terms of operating the system with the most suitable signal strength, which will correspond to the vehicle traveling speed on the track to meet high positioning accuracy. The objective in the next phase of work is to develop the two other subsystems, the data communication subsystem (DCS) and asset information subsystem (AIS), of the complete positioning system. This is since the testing of the physical demonstrator presented in this document addresses only the RFID subsystem. In addition, in this stage of work, only the standard single turnout is selected for development of lab-testing of the proposed positioning system. However, future work will carefully consider all the other types of junctions. Furthermore, it has not assessed potential issues due to long-term operation in real conditions such as a reflection from the vehicle body or nearby components; these will also be considered and tested in the next phase of the work. This will need further on-site trials to be carried out, where the RFID reader of this developed positioning system will be mounted on the TRV vehicle, and the RFID tags will be installed on the S&C system along the track.

## Figures and Tables

**Table 1:** Summary of the results and findings including the recommended signal strength to achieve a positioning accuracy less than ±1 m.

Technology	Speed Conditions [mph]		Reading [Yes/No]	Range of Signal Strength to Achieve a Positioning Accuracy Less than ±1 m
Passive RFID- Speedway (R420)	Low	5	Yes	50%
		10	Yes	
		20	Yes	50% to 60%
	Medium	25	Yes	65% to 70%
		35	Yes	70%
		50	Yes	70% to 75%
	High	70	Yes	80%, even when the presence of debris
		100	Yes	90%
		>120	Yes	100% (+31 dB)
Semi-passive RFID-TRANSIT(Ultimate) [Tag-ID DD000001]	Low	5	Yes	60%
		10	Yes	
		20	Yes	
	Medium	25	Yes	60% to 70%
		35	Yes	70% to 80%
		50	Yes	80% to 100%
	High	70	Yes	80% to 100% (+20 dB), even when the presence of debris
		>100	No	Tags could not be detected, even at 100% signal strength and 500 mm vertical height

**Table 2:** Summary of the outcomes/observations between the two RFID technologies against some testing parameters/variables.

Parameter/Variable	Passive RFID-Speedway	Semi-Passive RFID-TRANSIT
Detection/reading capability	Can detect and read the tag even up to max-speed (reach 140 mph)	Can detect and read the tag to a high speed of 70 mph. Could not detect and read the tag from a max-speed (>100 mph)
Positioning accuracy	Can provide a positioning accuracy less than ±1 m at all speed scenarios (with suitable strength of a signal shown in Table 1)	Can provide positioning accuracy less than ±1 m at all speed scenarios (with a suitable signal strength), except at max-speed scenario (>100 mph) where the tag could not be detected
Vertical height changes between the RFID reader and tag	It did not affect the detection/reading capability nor the positioning accuracy.	It did not affect the detection/ reading capability, but it did affect positioning accuracy. Larger height, less positioning accuracy (this could be improved by reducing the signal strength)
Presence of debris	It did not affect the detection/reading capability nor the positioning accuracy	
Ambient temperature and humidity variation within the same season (18 to 20.5 °C and 53 to 63%, respectively)		

**Table 3:** The evaluation summary of the passive RFID-Speedway and the semi-passive RFID-TRANSIT against the most important system requirements defined in the technical workshop and other important specifications defined after having installed and tested the technologies.

Top-Level Requirements		Values and Comments	Passive RFID-Speedway	Semi-Passive RFID- TRANSIT
Functional	Tag can be read also by a handheld device *	The secondary usage of the RFID system, which is also useful, is to provide on-site secure access to key asset information by maintenance personnel who should have a handheld device	Yes	No
	Passage speed	Between 5 and 70 mph	After being tested in the filed	
			Can be detected at speed over 100 mph that can be useful if this RFID system will be mounted on the New Measurement Train [35]	Up to 70 mph at a vertical distance of 400 ± 100 mm
	Battery life of the RFID tag	Passive or semi-passive (10 years lifetime)	Free-Battery	8 years lifetime
Data communication	Operating frequency	860 to 960 MHz, or 2.45 GHz	No clashing with other frequencies (like as WIFI- GSM)	Possibility of having interference of other equipment when it is working in the same frequency band
	Data communication platform development	Embedded system supports SDK	Yes	Might be impossible (No certain information from the supplier)
	Signal strength transmitted between the RFID tag and the reader	Possibility of changing the sensitivity or the power signal with software commands or codes	Yes	No (only possible with hardware manipulation)
Can the transceiver (reader) also write to a transponder (tag)? And are the tags re-writable? *		This is an important feature that allows good flexibility to locally re-program the tag on-site, without going back to the developer company each time need to store the tag-ID, to meet the corresponding S&C ID	Yes	No
Reader mounting place *		Reader should be in a safe place on-board. So, the risk of damaging the RFID system is low.	Yes, only the antenna needs to be mounted underneath the vehicle	No, the antenna is integrated with the reader that is needed to be mounted underneath the vehicle
Purchasing cost *		Tag	90% cheaper than TRANSIT Tag (A)	A
		Reader and Antenna	40% cheaper than TRANSIT unit (B)	B

* Other important specifications defined after having install and test the technologies.

## Data Availability

Not applicable.
